# Das neuartige Virus trifft auf die alten Verteilungsmechanismen: Warum die COVID-19-Pandemie zu mehr sozialer Ungleichheit führt

**DOI:** 10.1007/s10273-021-2817-5

**Published:** 2021-01-19

**Authors:** Christoph Butterwegge

**Affiliations:** Anton-Antweiler-Str. 24, 50937 Köln, Deutschland

## Abstract

Stößt das neuartige Coronavirus auf Menschen, deren ökonomische Lage und/oder sozialer Status sich deutlich unterscheiden, weichen die gesundheitlichen Auswirkungen für die Betroffenen häufig stark voneinander ab. Infektions-, Morbiditäts- und Mortalitätsrisiken der einzelnen Bevölkerungsschichten differieren zum Teil ganz erheblich, sind mit Abstand am höchsten bei armen und am niedrigsten bei reichen Personen. Die wirtschaftlichen Kollateralschäden der Pandemie und der Infektionsschutzmaßnahmen des Staates (zweimaliger bundesweiter Lockdown) verteilen sich ebenfalls nicht gleichmäßig über alle Bewohner:innen der Bundesrepublik. Vielmehr gibt es Gewinner:innen und Verlierer:innen, sowohl in der Wirtschaft (Differenzierung zwischen einzelnen Branchen) als auch in der Gesamtgesellschaft (Polarisierung zwischen verschiedenen Klassen und Schichten). Schließlich weisen die bisherigen Hilfsmaßnahmen, Finanzspritzen und Rettungsschirme des Staates eine verteilungspolitische Schieflage auf, wodurch die sozioökonomische Ungleichheit wächst, statt abgemildert zu werden.

Angesichts der bevorstehenden Konflikte um die Rückführung der hohen Staatsverschuldung, die Deutschland in den nächsten Jahren vor eine politische Zerreißprobe stellen dürften, sollte Verteilungsgerechtigkeit bei der Subventionsvergabe oberste Priorität haben. Wenn der Sozialstaat nicht in dem Sinne als „systemrelevant“ gelten will, dass er nur die bestehenden Herrschaftsverhältnisse, Machtstrukturen und Verteilungsmechanismen stabilisiert, muss er die Fehlkonstruktion der staatlichen Finanzhilfen zügig revidieren und künftig diejenigen Personengruppen stärker unterstützen, die auf den Märkten, insbesondere dem Arbeits- und dem Mietwohnungsmarkt, die geringsten Durchsetzungschancen haben.

Während der COVID-19-Pandemie ist die soziale Ungleichheit – wie unter einem Brennglas – deutlicher sichtbar geworden, hat sich durch die monatelange Krise aber auch drastisch verstärkt. Von einem „Ungleichheitsvirus" kann mit Blick auf SARS-CoV-2 jedoch ebenso wenig die Rede sein wie von einem sozialen Gleichmacher. Die tiefe Kluft zwischen Arm und Reich bestand vielmehr schon vor der Pandemie (Butterwegge, 2020a). Auch war das neuartige Coronavirus nicht für die gesellschaftlichen Rahmenbedingungen, das kapitalistische Wirtschaftssystem sowie die verteilungspolitischen Folgen der Entscheidungen des Staates zum Infektionsschutz und zur Bewältigung der Krisenfolgen verantwortlich.

Sucht man nach den Ursachen der seit Pandemiebeginn wachsenden Ungleichheit, lassen sich ein gesundheitlich oder pandemiebedingter Polarisierungsprozess, ein ökonomisch oder rezessionsbedingter Polarisierungsprozess und ein verteilungspolitisch oder subventionsbedingter Polarisierungsprozess voneinander unterscheiden, die im Folgenden analysiert werden.

## Potenzierung der gesundheitlichen Ungleichheit

Arbeitsbedingungen, Wohnverhältnisse und Gesundheitszustand (Zahl und Schwere der sozial bedingten Vorerkrankungen) üben einen signifikanten Einfluss auf das Infektions-, Morbiditäts- bzw. Mortalitätsrisiko der Bundesbürger:innen aus, die deshalb ganz unterschiedlich von der Pandemie betroffen sind. Hieß es früher aufgrund der je nach Geschlecht immer noch zehn Jahre höheren Lebenserwartung von Wohlhabenden und Reichen „Wer arm ist, muss früher sterben", so änderte sich diese Faustregel durch die Pandemie geringfügig: „Wer arm ist, muss eher sterben" heißt es jetzt, weil das Risiko, an COVID-19 zu sterben, für Wohlhabende und Reiche sehr viel niedriger ist.

Am härtesten trifft das Virus ausgerechnet die Immun- und die Finanzschwächsten, also zwei personell weitgehend identische Bevölkerungsgruppen. Arbeitslose, Abgehängte und Arme weisen häufiger als die übrigen Gesellschaftsmitglieder sozial bedingte Vorerkrankungen wie Adipositas (Fettleibigkeit), Asthma, Diabetes mellitus (Zuckerkrankheit) oder COPD (Raucherlunge) auf. Auch katastrophale Arbeitsbedingungen (z. B. in der Fleischindustrie) sowie beengte und hygienisch bedenkliche Wohnverhältnisse erhöhen das Risiko für eine Infektion mit dem neuartigen Coronavirus bzw. für einen schweren COVID-19-Krankheitsverlauf.

Überwiegend einkommens- und immunschwach waren Obdach- und Wohnungslose, aber auch andere Bewohner:innen von Gemeinschaftsunterkünften wie Strafgefangene, Geflüchtete, (süd)osteuropäische Werkvertragsarbeiter:innen der Subunternehmen deutscher Großschlachtereien bzw. Fleischfabriken und nichtdeutsche Saisonarbeiter:innen in der Landwirtschaft, außerdem Migrant:innen ohne gesicherten Aufenthaltsstatus, Menschen mit Behinderungen, Pflegebedürftige, Suchtkranke, Prostituierte, Erwerbslose, Geringverdiener:innen, Kleinstrentner:innen und Transferleistungsbezieher:innen (Empfänger:innen von Arbeitslosengeld II, Sozialgeld, Grundsicherung im Alter und bei Erwerbsminderung sowie Asylbewerberleistungen).

## Verstärkung der wirtschaftlichen Ungleichheit

Wie nie zuvor nach dem Zweiten Weltkrieg wurde im Gefolge des ersten Lockdowns im Frühjahr 2020 erkennbar, dass ein großer Teil der Bevölkerung trotz eines relativ hohen Lebens- und Sozialstandards sowie entgegen den Beteuerungen der politisch Verantwortlichen, die Bundesrepublik sei eine „klassenlose" Gesellschaft mit gesicherter Wohlständigkeit all ihrer Mitglieder, nicht einmal kurze Zeit ohne seine ungeschmälerten Regeleinkünfte auskommt (Butterwegge 2020b, 136 ff.).

Durch wochenlange Kontaktverbote, Ausgangsbeschränkungen und Einrichtungsschließungen wurde die ohnehin brüchige Lebensgrundlage der ärmsten Menschen (Bettler:innen, Pfandsammler:innen und Verkäufer:innen von Straßenzeitungen) zerstört, weil fehlende Passant:innen und die Furcht der verbliebenen davor, sich zu infizieren, manch-

**Prof. Dr. Christoph Butterwegge** lehrte von 1998 bis 2016 Politikwissenschaft in Köln und ist Mitglied der Forschungsstelle für interkulturelle Studien (FiSt) an der Universität zu Köln.

mal zum Totalausfall der Einnahmen führten, was stärkere Verelendungstendenzen in diesem Sozialmilieu nach sich zog. Die finanzielle Belastung von Transferleistungsbezieher :innen, Kleinstrentner:innen und Geflüchteten nahm durch die Schließung der meisten Lebensmitteltafeln, durch Hamsterkäufe geleerte Regale mit preiswerten Grundnahrungsmitteln und steigende Preise bei Frischeprodukten zu.

Die als Reaktion auf die Pandemie behördlich verordnete Schließung von Geschäften, Gaststätten, Hotels, Clubs, Kinos, Theatern und anderen Einrichtungen hatte erhebliche wirtschaftliche Einbußen für die Eigentümer:innen und die dort Tätigen, aber auch Konkurse und Entlassungen zur Folge. Einerseits blieben Kurzarbeit für Millionen Beschäftigte, Insolvenzen kleiner und mittlerer Unternehmen sowie Entlassungen (z.B. in der Gastronomie, der Touristik und der Luftfahrtindustrie) nicht aus, andererseits realisierten Großkonzerne krisenresistenter Branchen (z.B. Lebensmittel-Discounter, Drogeriemärkte, Versandhandel, Lieferdienste, Digitalwirtschaft und Pharmaindustrie) in der Corona-Krise sogar Extraprofite. Ob man ein Reisebüro oder einen Baumarkt besaß, der während des Lockdowns nicht geschlossen werden musste, machte einen riesigen Unterschied.

Zu den Hauptprofiteuren des Krisendesasters gehörten einige der profitabelsten Unternehmen mit den reichsten Eigentümer:innen. Unter dem Druck der Corona-Krise, die zu Einkommensverlusten durch Kurzarbeit, Geschäftsaufgaben und Arbeitslosigkeit geführt hat, kauften mehr Familien bei Lebensmittel-Discountern ein, um Geld zu sparen, wodurch die Inhaber von Ladenketten wie Aldi Nord und Aldi Süd, die ohnehin zu den vermögendsten Deutschen gehören, noch reicher geworden sein dürften. Dafür spricht die Entwicklung des Privatvermögens von Dieter Schwarz, dem Lidl und Kaufland gehören, das vor der Pandemie mit 41,5 Mrd. Euro veranschlagt wurde und laut Welt am Sonntag (vom 20.9.2020) im September dieses Jahres 41,8 Mrd. Euro betrug.

Viele kleine Einzelhändler:innen haben wegen der Schließung ihrer Läden und ausbleibender Kundschaft hingegen ihre Existenzgrundlage verloren. Wahrscheinlich hat sich die Kluft zwischen Arm und Reich nicht zuletzt deshalb am Ende weiter vertieft. Bei einer vom DIW unter den SOEP-Haushalten durchgeführten Ergänzungsbefragung berichteten jedenfalls 20% der im Jahr 2019 erwerbstätigen Personen, dass ihr Einkommen aufgrund der Corona-Krise gesunken sei: „Ein Verlust des Erwerbseinkommens wird zu einem Viertel etwas häufiger von den Erwerbstätigen im unteren bzw. oberen Terzil im Vergleich zum mittleren Terzil (ca. 16 Prozent) angegeben.“ (Schröder et al., 2020, 3)

Wie aus den Daten einer Panelbefragung im Auftrag der Hans-Böckler-Stiftung hervorgeht, gehörten Freiberufler:innen und Soloselbstständige, aber auch geringfügig oder befristet Beschäftigte und Leiharbeiter:innen zu den Hauptverlierer:innen der Corona-Krise, wobei die Kurzarbeit offenbar eine Schlüsselrolle spielte (Hövermann und Kohlrausch, 2020, 489). Auch zwischen einzelnen Beschäftigtengruppen nahm die Einkommensungleichheit während der durch die Pandemie ausgelösten Rezession zu. Insbesondere die unteren Einkommensbezieher:innen erlitten herbe Verluste, denn Geringverdiener:innen mussten sowohl häufiger wie auch stärker ins Gewicht fallende Einbußen hinnehmen (Kohlrausch et al., 2020).

Das vermehrte Homeoffice traf wegen des auftretenden Betreuungsproblems letztlich Frauen und Mütter, die hierdurch im Rahmen einer traditionellen Rollenverteilung zwischen den Geschlechtern wieder stärker zur Konzentration auf die Familienarbeit genötigt wurden. Zugenommen hat deshalb auch die Ungleichheit der Geschlechter, weil sich Beruf und Familie noch weniger miteinander vereinbaren ließen als sonst. Bettina Kohlrausch und Aline Zucco sprechen in diesem Zusammenhang von einer „doppelten“ Benachteiligung von Frauen durch weniger Erwerbseinkommen und mehr Sorgearbeit (Kohlrausch und Zucco, 2020).

### Verteilungspolitisch erzeugte Ungleichheit: Unsummen für die Wirtschaft – Brosamen für die Armen?

Bund, Länder und Gemeinden haben in der Corona-Krise nach kurzem Zögern riesige Hilfspakete geschnürt, die aus Finanzspritzen, Bürgschaften und Krediten bestanden. Letztere kamen in erster Linie großen Unternehmen zugute, während kleine und mittlere Firmen mit Zuschüssen unterstützt wurden, die laufende Betriebskosten decken, aber von Soloselbstständigen nicht zur Bestreitung ihres Lebensunterhalts verwendet werden sollten. Während zahlreiche Unternehmen, darunter auch solche mit einer robusten Kapitalausstattung, von der Bereitschaft des Staates zu einer hohen Neuverschuldung profitierten, kamen die sozial Benachteiligten eher zu kurz.

Zu den von der Pandemie finanziell stark geschädigten Gruppen gehörten die Beschäftigten im Niedriglohnsektor. „Wie keine andere Beschäftigtengruppe werden sie mit Kurzarbeit konfrontiert und müssen entsprechende Einkommenseinbußen hinnehmen. Dabei greifen sozialpolitische Kompensationsmaßnahmen wie die Aufstockung des Kurzarbeitergeldes gerade für diese Beschäftigtengruppe am wenigsten.“ (Schulten, 2020, 16) Das bei einer um mindestens 50% reduzierten Arbeitszeit nach dreimonatigem Bezug auf 70% bzw. 77% und nach sechsmonatigem Bezug auf 80% bzw. 87% angehobene Kurzarbeitergeld schützt sie nicht vor Armut, wohingegen ihnen ein Mindestkurzarbeitergeld mehr nützen würde als die von CDU, CSU und SPD vorgenommenen Verbesserungen (Einbeziehung der Leiharbeiter:innen und Erweiterung der Hinzuverdienstmöglichkeiten für Kurzarbeiter:innen).

Überstundenzuschläge, Einmalzahlungen (z.B. Gewinnbeteiligungen oder Jahresprämien) sowie steuer- und beitragsfreie Zuschläge für Sonntags-, Feiertags- und Nachtarbeit bleiben bei der Berechnung des Kurzarbeitergeldes unberücksichtigt, was im Falle der „Kurzarbeit Null" nicht bloß für Geringverdiener:innen drastische Einbußen gegenüber ihrem Lohn und gravierende Einschränkungen ihres gewohnten Lebensstandards mit sich brachte. Beschäftigte aus bessersituierten Haushalten sind dagegen seltener von Kurzarbeit betroffen (Kruppe und Osiander, 2020, 6).

Studierenden, die häufig ihren Nebenjob (z.B. in der Gastronomie) verloren, der ihren Lebensunterhalt bis dahin gesichert hatte, stand weder Kurzarbeiter- noch Arbeitslosengeld (I bzw. II) zu, was manchmal den Studienabbruch zur Folge hatte. Nothilfe des Bundes in Höhe von maximal 500 Euro können Studierende nur erhalten, wenn sie weniger als diesen Betrag auf ihrem Konto haben. Umso großzügiger war der Staat bei den Großkonzernen: BMW ließ sich für 30.000 Beschäftigte in Kurzarbeit fast die gesamten Lohnkosten (einschließlich der Arbeitgeberbeiträge zur Kranken-, Pflege- und Rentenversicherung) erstatten, schüttete aber im Mai 2020 nicht weniger als 1,64 Mrd. Euro an Dividenden für das Vorjahr aus. Davon erhielten die Großaktionäre Susanne Klatten und Stefan Quandt, denen fast die Hälfte des Münchener Automobilherstellers gehört, allein 769 Mio. Euro. Frankreich, Dänemark und Schweden machen es Unternehmen in einem solchen Fall zur Auflage, keine Gewinne auszuschütten.

Die am meisten beachtete Maßnahme des Konjunktur- und Krisenbewältigungspakets der Großen Koalition war die zeitweilige Senkung des Mehrwertsteuersatzes von 19 % auf 16 % bzw. von 7 % auf 5 %. Je umsatzstärker ein Unternehmen war, umso stärker profitierte es von der Mehrwertsteuersenkung, erst recht natürlich dann, wenn es diese nicht an seine Kundschaft weitergab. Bei der Ausweitung des steuerlichen Verlustrücktrags, der Einführung einer degressiven Abschreibung für Abnutzung (AfA) mit einem höheren Faktor und maximal 25 % pro Jahr für bewegliche Wirtschaftsgüter des Anlagevermögens 2020/2021 und der unbefristeten „Modernisierung" des Körperschaftsteuerrechts (Einführung eines Optionsmodells zur Körperschaftsteuer für Personengesellschaften) handelte es sich noch weniger um passgenaue Hilfen, sondern um reine Steuergeschenke, die der Wirtschaftsflügel von CDU und CSU den Unternehmern schon lange machen wollte. Ähnliches gilt für die „Sozialgarantie 2021", mit der die Sozialversicherungsbeiträge durch den Einsatz von Steuermitteln bei 40 % vom Bruttolohn oder -gehalt gedeckelt und steigende „Lohnnebenkosten" – gemeint sind die Arbeitgeberbeiträge zur Sozialversicherung – verhindert werden sollen.

Auch die „November-" und „Dezemberhilfen" während des zweiten (Teil-)Lockdowns haben zur Überprivilegierung der Kapitaleigentümer:innen beigetragen. Weil der Bund seinen Kompensationszahlungen pauschal 75 % des im entsprechenden Vorjahresmonat erzielten Umsatzes statt des realen Betriebsergebnisses zugrunde legte, stellten sich manche Unternehmer:innen zeitweilig sogar besser als vor der Pandemie. In der staatlichen Subventionspraxis schlug sich die wirtschaftsliberale Ideologie nieder, nach der ein Unternehmer per se als „Leistungsträger" der Gesellschaft gilt, wohingegen sozial benachteiligte Personengruppen eher zu den Leistungsverweigerern gezählt werden.

## Zusammenfassung und Schlussfolgerungen

Stößt das neuartige Coronavirus auf Menschen, deren ökonomische Lage und/oder sozialer Status sich deutlich unterscheiden, weichen die gesundheitlichen Auswirkungen für die Betroffenen häufig stark voneinander ab. Infektions-, Morbiditäts- und Mortalitätsrisiken der einzelnen Bevölkerungsschichten differieren zum Teil ganz erheblich, sind mit Abstand am höchsten bei armen und am niedrigsten bei reichen Personen. Die wirtschaftlichen Kollateralschäden der Pandemie und der Infektionsschutzmaßnahmen des Staates (zweimaliger bundesweiter Lockdown) verteilen sich ebenfalls nicht gleichmäßig über alle Bewohner:innen der Bundesrepublik. Vielmehr gibt es Gewinner:innen und Verlierer:innen, sowohl in der Wirtschaft (Differenzierung zwischen einzelnen Branchen) als auch in der Gesamtgesellschaft (Polarisierung zwischen verschiedenen Klassen und Schichten). Schließlich weisen die bisherigen Hilfsmaßnahmen, Finanzspritzen und Rettungsschirme des Staates eine verteilungspolitische Schieflage auf, wodurch die sozioökonomische Ungleichheit wächst, statt abgemildert zu werden.

Angesichts der bevorstehenden Konflikte um die Rückführung der hohen Staatsverschuldung, die Deutschland in den nächsten Jahren vor eine politische Zerreißprobe stellen dürften, sollte Verteilungsgerechtigkeit bei der Subventionsvergabe oberste Priorität haben. Wenn der Sozialstaat nicht in dem Sinne als „systemrelevant" gelten will, dass er nur die bestehenden Herrschaftsverhältnisse, Machtstrukturen und Verteilungsmechanismen stabilisiert, muss er die Fehlkonstruktion der staatlichen Finanzhilfen zügig revidieren und künftig diejenigen Personengruppen stärker unterstützen, die auf den Märkten, insbesondere dem Arbeits- und dem Mietwohnungsmarkt, die geringsten Durchsetzungschancen haben.
